# Modeling Cell Gradient Sensing and Migration in Competing Chemoattractant Fields

**DOI:** 10.1371/journal.pone.0018805

**Published:** 2011-04-29

**Authors:** Dan Wu, Francis Lin

**Affiliations:** 1 Department of Physics and Astronomy, University of Manitoba, Winnipeg, Canada; 2 Department of Immunology, University of Manitoba, Winnipeg, Canada; 3 Department of Biological Sciences, University of Manitoba, Winnipeg, Canada; 4 Department of Biosystems Engineering, University of Manitoba, Winnipeg, Canada; Duke University Medical Center, United States of America

## Abstract

Directed cell migration mediates physiological and pathological processes. In particular, immune cell trafficking in tissues is crucial for inducing immune responses and is coordinated by multiple environmental cues such as chemoattractant gradients. Although the chemotaxis mechanism has been extensively studied, how cells integrate multiple chemotactic signals for effective trafficking and positioning in tissues is not clearly defined. Results from previous neutrophil chemotaxis experiments and modeling studies suggested that ligand-induced homologous receptor desensitization may provide an important mechanism for cell migration in competing chemoattractant gradients. However, the previous mathematical model is oversimplified to cell gradient sensing in one-dimensional (1-D) environment. To better understand the receptor desensitization mechanism for chemotactic navigation, we further developed the model to test the role of homologous receptor desensitization in regulating both cell gradient sensing and migration in different configurations of chemoattractant fields in two-dimension (2-D). Our results show that cells expressing normal desensitizable receptors preferentially orient and migrate toward the distant gradient in the presence of a second local competing gradient, which are consistent with the experimentally observed preferential migration of cells toward the distant attractant source and confirm the requirement of receptor desensitization for such migratory behaviors. Furthermore, our results are in qualitative agreement with the experimentally observed cell migration patterns in different configurations of competing chemoattractant fields.

## Introduction

Migratory responses of cells to cellular guiding signals play important roles in regulating a wide range of physiological and pathological processes such as inflammation and autoimmune diseases [1], wound healing [2], [3], neuron guidance [4], [5], embryogenesis [6], and cancer metastasis [7], [8]. In particular, chemoattractant gradients guide the migration of immune cells (i.e., chemotaxis), orchestrating cell trafficking and positioning in tissues [9], [10]. It has been shown that leukocytes express multiple different chemoattractant receptors in a cell subset dependent manner, and can integrate multiple co-existing chemotactic signals to direct their migration to specific targets in tissues that enable immune surveillance and immune responses [9], [11]. Experimental studies of neutrophil migration reveal that cells preferentially migrate toward a distant chemoattractant source in two competing chemoattractant gradients [12], [13], [14], [15], [16]. The higher sensitivity of cells to the distant attractant source suggests a multi-step model wherein cells navigate through an array of chemoattractant sources in a step-by-step manner (i.e., multi-step chemotactic navigation) [12], [13]. However, the underlying mechanism for chemotactic signal integration and multi-step chemotactic navigation is not clearly defined.

Previous modeling and experimental studies have investigated gradient sensing and chemotaxis in single chemoattractant gradients [17], [18], [19]. For eukaryotic cells, chemoattractant receptors are uniformly distributed on the cell surface and bind to chemoattractant molecules to initiate downstream chemotactic signaling [20]. It has been well accepted that chemoattractant receptor occupancy difference across the cell length is a determining factor for cell gradient sensing and migration while the robustness of gradient sensing and chemotaxis in shallow chemoattractant gradients is enabled by downstream signal amplification and adaptation mechanisms [21]. Simplistic models based on receptor-ligand binding have been previously developed for cell orientation and migration [22]. Furthermore, ligand-induced homologous receptor desensitization is a conserved property for all G-protein coupled chemoattractant receptors and thus can regulate the number of signaling receptors for gradient sensing [18], [19]. The kinetic parameters for ligand-induced receptor modulations have been experimentally measured for human neutrophil formyl peptide receptors and receptor desensitization has been taken into account for modeling cell gradient sensing in single ligand gradients [23], [24], making it interesting for further modeling in multiple co-existing gradient fields.

A previous modeling study examined the role of ligand-induced homologous receptor desensitization in mediating cell gradient sensing [25]. The model considers the rapid deactivation of chemoattractant receptor signaling upon ligand binding and the subsequent receptor recycling, and shows that the preferred cell orientation toward the distant ligand gradient over the local competing ligand gradient is critically enabled by receptor desensitization. This study serves as the first step toward defining the cellular mechanism for chemotactic navigation and positioning of cells in complex ligand fields. Because this previous model is limited to cell gradient sensing at steady state in one-dimension (1-D), which is fundamentally different from the dynamic cell migration process, the modeling results could not be directly compared with experimental cell migration data. To overcome this limitation, in the present study, we further develop the model to test the role of ligand-induced homologous receptor desensitization for mediating cell gradient sensing in two-dimensional (2-D) ligand fields, and we performed computer simulations for the dynamic cell migration process in different configurations of ligand gradient fields. Consistent with the previous 1-D model, the 2-D modeling results show that cells expressing nondesensitizable receptors orient toward ligand gradients more robustly compared to cells expressing desensitizable receptors. In competing ligand gradients, cells expressing desensitizable receptors but not nondesensitizable receptors preferentially orient and migrate toward the distant ligand gradient. In addition, computer simulation data are in qualitative agreement with previous experimental results of neutrophil migration in different competing chemoattractant gradients and show that the different migration patterns are receptor desensitization dependent. Thus, our results not only validate the previous model in 2D gradients but also extend the importance of the receptor desensitization mechanism from gradient sensing to cell migration and trafficking in complex gradient environments.

## Methods

### Gradient Sensing Model in 2-D Ligand Fields

**Figure 1:**
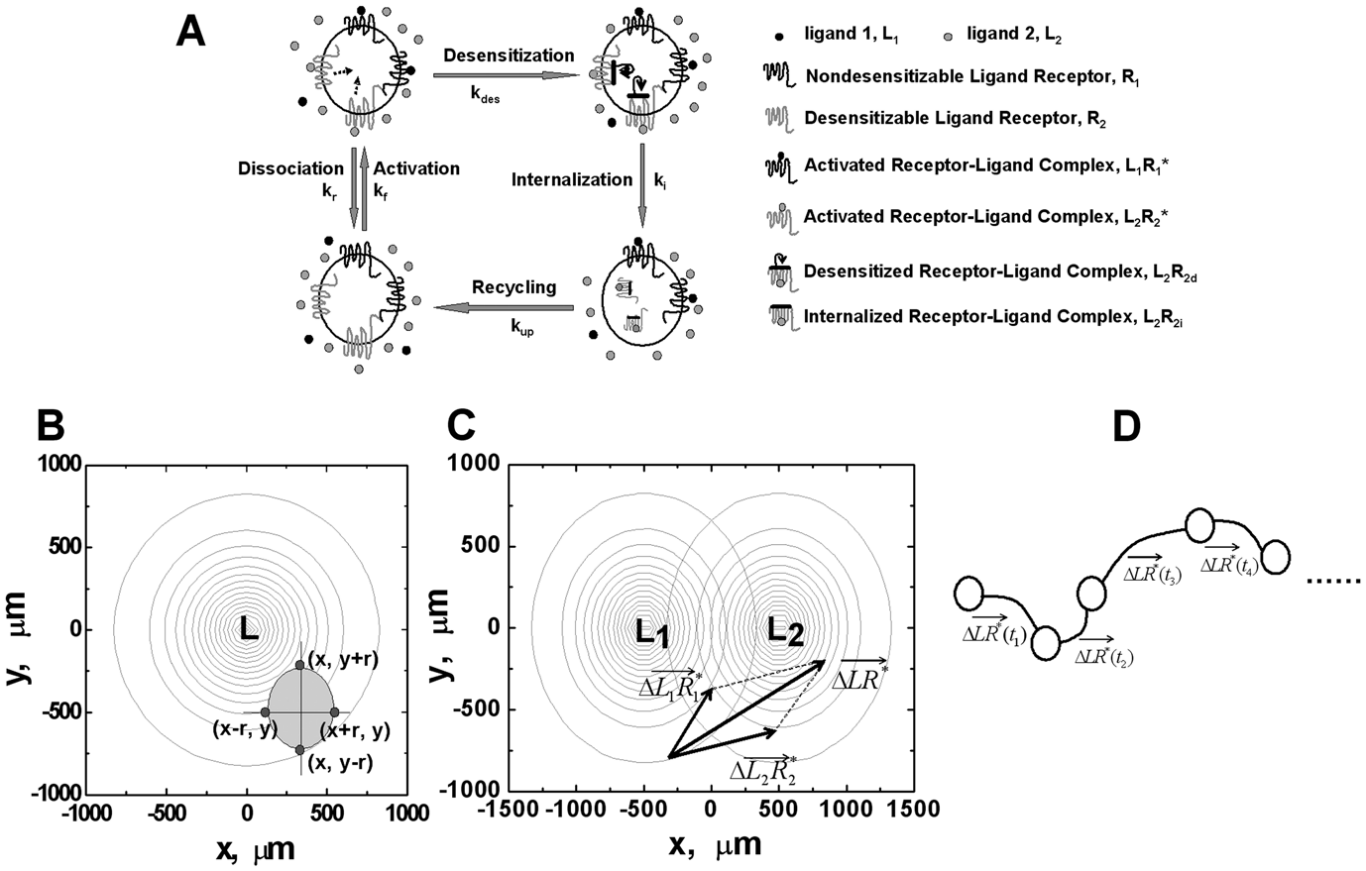
Schematic illustration of the model. (A) Receptors are initially expressed on the cell surface. Upon binding to the ligand molecules, the receptors are activated and trigger chemotactic signaling. Activated receptor-ligand complexes are rapidly deactivated (i.e., desensitization) which are subsequently internalized and eventually re-expressed back to the cell surface. (B) The cell is modeled as four receptor expressing units symmetrically located along the x and y axis with equal distance to the center of mass of the cell. In a single ligand gradient, the active receptor-ligand complex LR^*^ is evaluated for all four receptor expressing units of the cell, and the difference of LR^*^ along the x and y axis is calculated to determine the orientation strength in the two directions. The net orientation of the cell is determined by the orientation vector 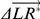 in the 2-D plane. (C) In competing gradients of two ligands *L_1_* and *L_2_*, the orientation vector of the cell to each ligand is evaluated and the net orientation vector of the cell is determined by the addition of the orientation vectors to *L_1_* and *L_2_*. (D) The model cell is allowed to move along the direction set by the net orientation vector 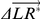. The migration step time is set to be directly proportional to the magnitude of the orientation vector with the optimal step time of 2.5 minutes. The migration speed is set at 10 µm/min. Within each migration step, the cell turns from its previous migration direction to the new direction set by the orientation vector at the turning rate directly proportional to the magnitude of the orientation vector through multiple sub-steps.

As illustrated in Figure 1A, we adapt the previous 1-D gradient sensing model to describe receptor-ligand binding, receptor desensitization and recycling. Briefly, receptors are initially expressed on the cell surface. Upon binding to the ligand molecules, the receptors are activated and trigger chemotactic signaling. Activated receptor-ligand complexes are rapidly deactivated (i.e., desensitization) and the desensitized receptors are subsequently internalized and eventually re-expressed back to the cell surface. Consistent with previous models, the dissociation of ligand from desensitized receptors on the cell surface is assumed negligible [23].

The model cell is simplified to consist of four receptor expressing units symmetrically located along the x and y axis with equal distance to the center of mass of the cell (r = 5 µm assuming the typical 10 µm diameter of neutrophils, [26]) (Figure 1B). This 4-unit cell model allows evaluation of cell orientation in 2D ligand gradient fields. We compared the cell orientation based on the 4-unit cell model to the cell model with more continuous receptor distribution on the cell surface (e.g. 36-unit, 72-unit, etc) and we found negligible difference (i.e. less than 0.03%). Therefore, the 4-unit cell model is sufficient to predict cell orientation in our model and minimizes the computation.

The time-dependent ligand induced receptor modulation at each receptor expressing unit is described by a system of ordinary differential equations [25]. The symbols for variables and kinetic rates in the model are defined in Table 1 and the values of kinetic rates and other parameters are adapted from the literature based on human neutrophil formyl peptide receptors [23], [24], [25], [27].
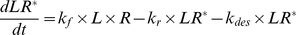
(1)

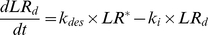
(2)

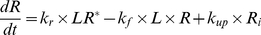
(3)The differential equations are subject to restraint conditions assuming total receptor conservation and that all receptors are initially expressed on the cell surface in the free receptor state.
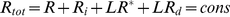
(4)

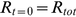
(5)

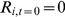
(6)

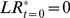
(7)

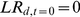
(8)Fixed nonlinear ligand gradients in a 2-D system are set up in the model (Figure 1B), so it is consistent with the previous 1-D model. The symbols are defined in Table 1.
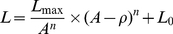
(9)The selection of this power gradient has been justified previously [25]. The power gradient (n = 3) provides a simple and fixed nonlinear ligand gradient profile for the model. In addition, the more realistic gradient profile from fixed point-source free diffusion can be effectively fitted by the power function with the power n = 2.9 at t = 3 min for 10 kDa chemokine molecules in medium (File S1). Furthermore, in this paper, we compared our modeling data to the experimental data in the under agarose assay [12], [13] and the gradient profile of LTB4 in the under agarose assays at 30 min can be effectively fitted by the power function with the power n = 3.35 (File S1). Thus, in the present study, we used this simple fixed power gradient with n = 3 for our model.

**Table 1:** Variables and parameters in the model.

Symbols	Implications	Values
*L, L_1_, L_2_*	ligand concentration	Variable (in nM)
*R, R_1_, R_2_*	number of free surface receptors	Variable
*R_i_, R_1i_, R_2i_*	number of intracellular free receptors	Variable
LR^*^, L_1_R_1_^*^, L_2_R_2_^*^	number of active receptor-ligand complex	Variable
*LR_d_, L_1_R_1d_, L_2_R_2d_*	number of desensitized receptor-ligand complex	Variable
*LR_i_, L_1_R_1i_, L_2_R_2i_*	number of internalized receptor-ligand complex	Variable
*R_tot_*	total number of receptors	25,000 [25]
*k_f_*	ligand receptor association rate	8.4×10^7^ M^−1^ s^−1^ [23], [35]
*k_r_*	low-affinity ligand receptor dissociation rate	0.37 s^−1^ [23], [27]
*k_des_*	desensitization rate	0.065 s^−1^ for desensitizable receptor[23], [27]; 0 for nondesensitizable receptor
*k_i_*	internalization rate	0.0033 s^−1^ [23], [36]
*k_up_*	up-regulation rate	0.004 s^−1^ [23], [24]
*L_max_, L_1max_, L_2max_*	highest concentration at the gradient center	17.6 nM [25] if no additional caption
*L_0_*	basal ligand concentration	0 nM [25]
*A*	radius of the gradient region	1000 µm [25]
*ρ, ρ_1_, ρ_2_*	distance from the gradient center	Variable (in µm) [25]
*n*	power of the gradient function	3 [25]

The numerical values for parameters are the same for both ligand-receptor pairs unless stated otherwise.

In a single ligand gradient, the active receptor-ligand complex LR^*^ is evaluated for all four receptor expressing units of the cell, and the difference of LR^*^ along the x and y axis is calculated to determine the orientation strength in the two directions. The net orientation of the cell is determined by the orientation vector 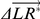 in the 2-D plane.
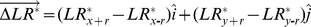
(10)In competing gradients of two ligands *L_1_* and *L_2_*, Eqs (1–9) are applied for *L_1_* and *L_2_*, and the notations in the model are adjusted accordingly with the index indicating specific ligand fields and receptors, i.e., L_1_R_1_*, L_2_R_2_*, *L_1_R_1d_*, *L_2_R_2d_*, *R_1_*, *R_2_*, *R_1i_*, *R_2i_*, *L_1max_*, *L_2max_*, *ρ_1_* and *ρ_2_*. The orientation vector of the cell to each ligand is evaluated as:
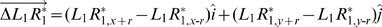
(11)

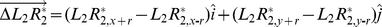
(12)The net orientation vector of the cell is determined by the addition of the orientation vectors to *L_1_* and *L_2_* (Figure 1C).
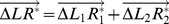
(13)In principle, different ligands and receptors can have different recycling kinetics and can trigger different downstream chemotactic signaling pathways [28], [29]. In addition, the profiles of ligand fields can be different depending on the diffusion properties of the ligands and their interactions with tissues [13]. Our model is simplified in that the two ligand-receptor pairs share the same kinetic properties and downstream signaling pathways unless stated otherwise (e.g., different desensitization rates in some cases). This allows us to focus on the role of receptor desensitization in gradient sensing and migration. The gradient profiles are set to be identical for *L_1_* and *L_2_* with different locations of *L_1max_*, and *L_2max_*. In addition, the total receptor numbers are assumed to be the same for *R_1_* and *R_2_*. The effect of differential receptor numbers on cell gradient sensing and migration has been discussed in previous studies and is not the focus of the current paper.

### Migration Model in 2-D Ligand Gradients

Based on the gradient sensing model, the model cell is allowed to move along the direction set by the net orientation vector. Initially, cells are located at different positions in the gradient fields to start migrating. The gradient sensing model as described in the previous section is applied to cells to determine their net orientation vector. The migration speed is set at 10 µm/min similar to the previously reported migration speed [30]. The differential equations are integrated by the 4th order Runge-Kutta algorithm. Consistent with the previous models [21], [22], [25], the threshold magnitude of the orientation vector for chemotactic orientation is set at 10, i.e., |ΔLR*|≥10. The setting of the threshold in our model is based on considerations of the minimal receptor occupancy difference required for cells to detect a ∼1% ligand concentration difference across the cell length [21], [22]. Below the threshold, i.e., |ΔLR*|<10, the cell orients and migrates randomly in the 2-D plane. The random orientation in the 2-D plane (0∼2π) is determined by a random number generator. The step length of persistent cell migration time is set to be directly proportional to the magnitude of the orientation vector with the optimal step length of 2.5 minutes, which is similar to the previously reported characteristic time of persistent cell migration [22]. The orientation vector is continuously evaluated and determines the direction of cell migration at each migration step (Figure 1D). Within each migration step, the cell turns from its previous migration direction to the new direction set by the orientation vector at the turning rate directly proportional to the magnitude of the orientation vector through multiple sub-steps.

Based on the model established above, we tested the role of ligand-induced homologous receptor desensitization in mediating cell gradient sensing at the steady state (*d* 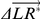 */dt = 0*) and the dynamic migratory behaviors of cells in single ligand gradients and in multiple competing ligand gradients in 2-D environments.

## Results

### Cell Orientation and Migration in Single Ligand Gradients

**Figure 2:**
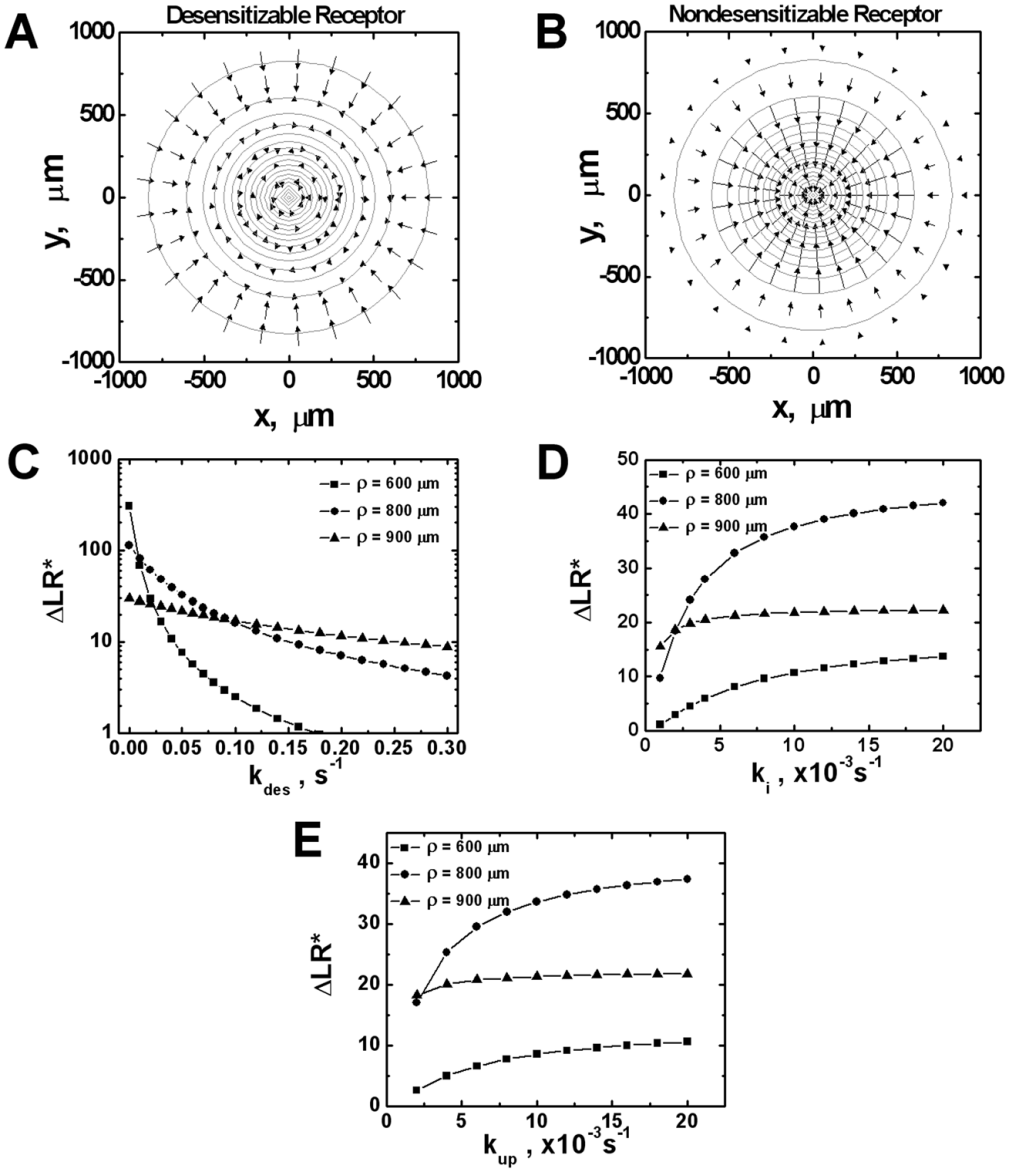
Comparison of orientation of cells expressing desensitizable and nondesensitizable receptors in a single ligand gradient. (A) Desensitizable receptors; (B) Nondesensitizable receptors. The cell orientation is represented by arrows in the figures and the length of the arrows indicates the strength of the orientation. The ligand gradient is represented by contour plot with the highest ligand concentration (17.6 nM) at the center of the contours for each gradient. The ligand concentration at the outmost contour circle is 0.1 nM, and the concentration difference between adjacent circles is 1.0 nM. Because of the magnitude difference between the orientation vector of cells expressing desensitizable and nondesensitizable receptors, the length of the arrow is adjusted with a scaling factor of 1.2 for desensitizable receptors, and 0.1 for nondesensitizable receptors. (C–E) The dependence of cell orientation strength (ΔLR^*^) on desensitization rate *k_des_* (C), internalization rate *k_i_* (D) and up-regulation rate *k_up_* (E) for cells locates at ρ = 600 µm, 800 µm and 900 µm. ρ is defined in Equation 9 and Table 1. Unless indicated otherwise, *k_des_* is fixed at 0.065 s^−1^, *k_up_* at 0.004 s^−1^ and *k_i_* at 0.0033 s^−1^.

We first compare chemotactic orientation of cells expressing desensitizable receptors or nondesensitizable receptors at steady state. As shown in Figure 2A, cells expressing desensitizable receptors orient toward the ligand gradient in the outer region of the ligand field where the ligand concentration is low. As the cells are closer to the ligand source where the ligand concentration is higher, the cells orient randomly (i.e., |ΔLR*|<10) resulting from receptor occupancy saturation and higher level of receptor desensitization. In contrast, cells expressing nondesensitizable receptors are able to orient toward the ligand gradient in almost the entire ligand field (Figure 2B), suggesting higher level of chemotactic orientation. The length of the arrow depicted in Figure 2 is proportional to the magnitude of the orientation vector and is scaled for visualization. Because of the magnitude difference between the orientation vector of cells expressing desensitizable and nondesensitizable receptors, the length of the arrow is adjusted with a scaling factor of 1.2 for Figure 2A, and 0.1 for Figure 2B.

We further performed parametric analysis to test the model at the steady state for a range of values of different kinetic rates (Figure 2C–2E). A range of positions in the gradient (i.e. ρ = 600 µm, 800 µm and 900 µm) are selected to evaluate the orientation vector ΔLR* of the cell at these positions over a range of desensitization rate *k_des_* (Figure 2C), internalization rate *k_i_* (Figure 2D) and up-regulation rate *k_up_* (Figure 2E). As expected, the strength of cell orientation toward the gradient (i.e. ΔLR*) decreases with increasing *k_des_*, and the specific dependence of cell orientation on *k_des_* varies with the cell position in the gradient field (Figure 2C). In contrast, cell orientation toward the gradient increases with increasing *k_i_* (Figure 2D) or *k_up_* (Figure 2E), and again the specific dependence of cell orientation on *k_i_* or *k_up_* also varies with the cell position in the gradient field.

**Figure 3:**
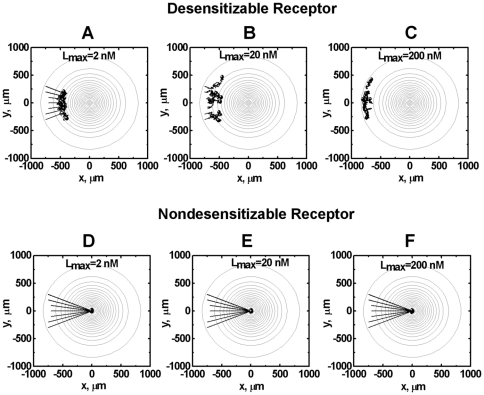
Comparison of migration of cells expressing desensitizable and nondesensitizable receptors in different single ligand gradients. (A–C) Desensitizable receptors; (D–F) Nondesensitizable receptors. The highest ligand concentration at each gradient center is 2 nM (A, D), 20 nM (B, E) and 200 nM (C, F). Seven representative cell tracks are shown for each simulation. The total time of simulated cell migration is 150 minutes, and the end of the tracks is indicated by solid circles.

Consistent with the orientation results in single ligand gradients, simulations of cell migration show higher level of chemotaxis for cells expressing nondesensitizable receptors compared to cells expressing desensitizable receptors. As demonstrated in Figure 3A–3C, cells expressing desensitizable receptors migrate more toward the ligand source when the maximum ligand concentration is relatively low (i.e., 2 nM and 20 nM) compared to the ligand gradient with high maximum concentration at the center of the gradient field (i.e., 200 nM). The cells could not migrate further as they reached the region where the ligand concentration is sufficiently high to lower the orientation vector below the threshold. In comparison, cells expressing nondesensitizable receptors migrate toward the ligand gradients in a dose-independent manner and are able to reach the ligand source without interruption of receptor signaling.

The results of cell orientation and migration in single ligand gradients validate the current 2-D model and are consistent with both the previous 1-D model [25] and experimental studies showing comparable or even higher levels of chemotaxis of cells mediated by nondesensitizable receptor mutants [18], [19].

### Cell Orientation and Migration in Competing Ligand Gradients

**Figure 4:**
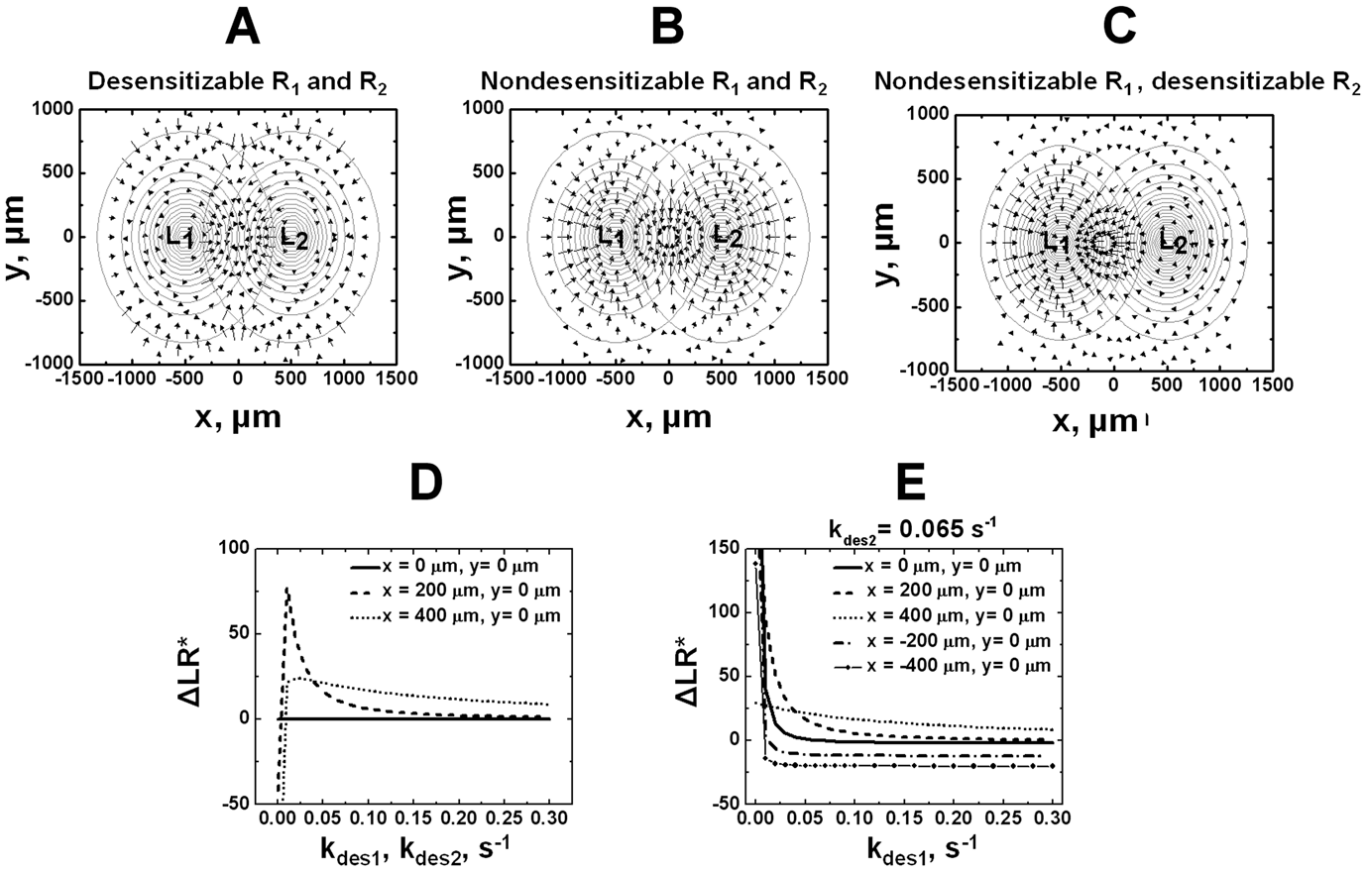
Cell orientation in competing ligand gradients at the steady state. (A) In competing gradients of *L_1_* and *L_2_*, cells expressing normal desensitizable receptors for both ligands orient toward the distant ligand gradient. i.e., cells that are close to *L_1_* orient toward *L_2_* and cells that are close to *L_2_* orient toward *L_1_*. (B) Cells expressing nondesensitizable receptors for both ligands orient toward the local ligand gradient. i.e., cells that are close to *L_1_* orient toward *L_1_* and cells that are close to *L_2_* orient to *L_2_*. (C) Cells expressing nondesensitizable receptor for *L_1_* but desensitizable receptor for *L_2_* orient toward the nondesensitizing ligand gradient *L_1_*. This effect is clear in the region where the two ligand gradients are significantly overlapped. The ligand gradients are represented by contour circles with the highest ligand concentration (17.6 nM) at the center of the contours for each gradient. The ligand concentration at the outmost contour circle is 0.1 nM, and the concentration difference between adjacent circles is 1.0 nM. Because of the magnitude difference between the orientation vector of cells expressing desensitizable and nondesensitizable receptors, the length of the arrow is adjusted, i.e., the scaling factor is set at 0.8 for Figure 4A, and 0.07 for Figure 4B and 4C. (D–E) The dependence of cell orientation (ΔLR^*^) on *k_des1_* and *k_des2_* for *L_1_* receptor and *L_2_* receptor respectively that vary at the same time (D), or on *k_des1_* for *L_1_* receptor (E), for cells locates at different positions in the overlapping area of *L_1_* and *L_2_*. Positive ΔLR^*^ indicates cell orientation toward *L_1_* and negative ΔLR^*^ indicates cell orientation toward *L_2_*.

Given the results that nondesensitizable receptors mediate cell orientation and migration in single ligand gradients at a higher level, we further test the influence of receptor desensitization on gradient sensing and migration in competing ligand gradients. Initially, we compare cell orientation at steady state in competing ligand gradients mediated by desensitizable or nondesensitizable receptors. As shown in Figure 4A, desensitizable receptors allow cells to orient toward the distant ligand source in the region where two ligand gradients are overlapped oppositely. In contrast, cells expressing nondesensitizable receptors in the same overlapping gradient region orient toward the local nearer ligand source (Figure 4B). In both cases, cells lose orientation toward either ligand source in the central region of competing ligand gradients where the receptor signaling to the two ligand gradients is balanced. However, the random orientation of cells expressing desensitizable receptors is stabilized in this region whereas this inability of gradient sensing is unstable for cells expressing nondesensitizable receptors and the cells will eventually migrate away from the central region toward the nearer ligand source. In the scenario that cells express desensitizable receptor for one ligand, but nondesensitizable receptor for the other ligand, the cells orient toward the nondesensitizing ligand source because of a stronger chemotactic signal (Figure 4C). Again, because of the magnitude difference between the orientation vector of cells expressing desensitizable and nondesensitizable receptors, the length of the arrow is adjusted, i.e., the scaling factor is set at 0.8 for Figure 4A, and 0.07 for Figure 4B and 4C.

Similar to the analysis in single ligand gradients, we further performed parametric analysis to test the model at the steady state for a range of values of different kinetic rates (Figure 4D&4E). In competing gradients of 2 different ligands, the cell adjusts its orientation from toward the local ligand gradient to toward the distant ligand gradient with increasing receptor desensitization rate (assume both *k_des1_* and *k_des2_* vary at the same time) and the cell will eventually lose any chemotactic orientation as the receptor desensitization rate keeps increasing (Figure 4D). Consistently, if only varies the desensitization level of one receptor (*k_des1_*) and keeps the desensitization rate of the other receptor a constant (*k_des2_ = 0.065 s^−1^*), the cell initially locates close to *L_1_* will adjusts its orientation from toward *L_1_* to toward *L_2_* with increasing *k_des1_* (Figure 4E). Similarly, the cell initially locates close to *L_2_* will adjusts its orientation from toward *L_1_* to random orientation with increasing *k_des1_* (Figure 4E). The specific dependence of cell orientation on *k_des_* varies with the relative cell position in the gradient fields (Figure 4D&4E).

**Figure 5:**
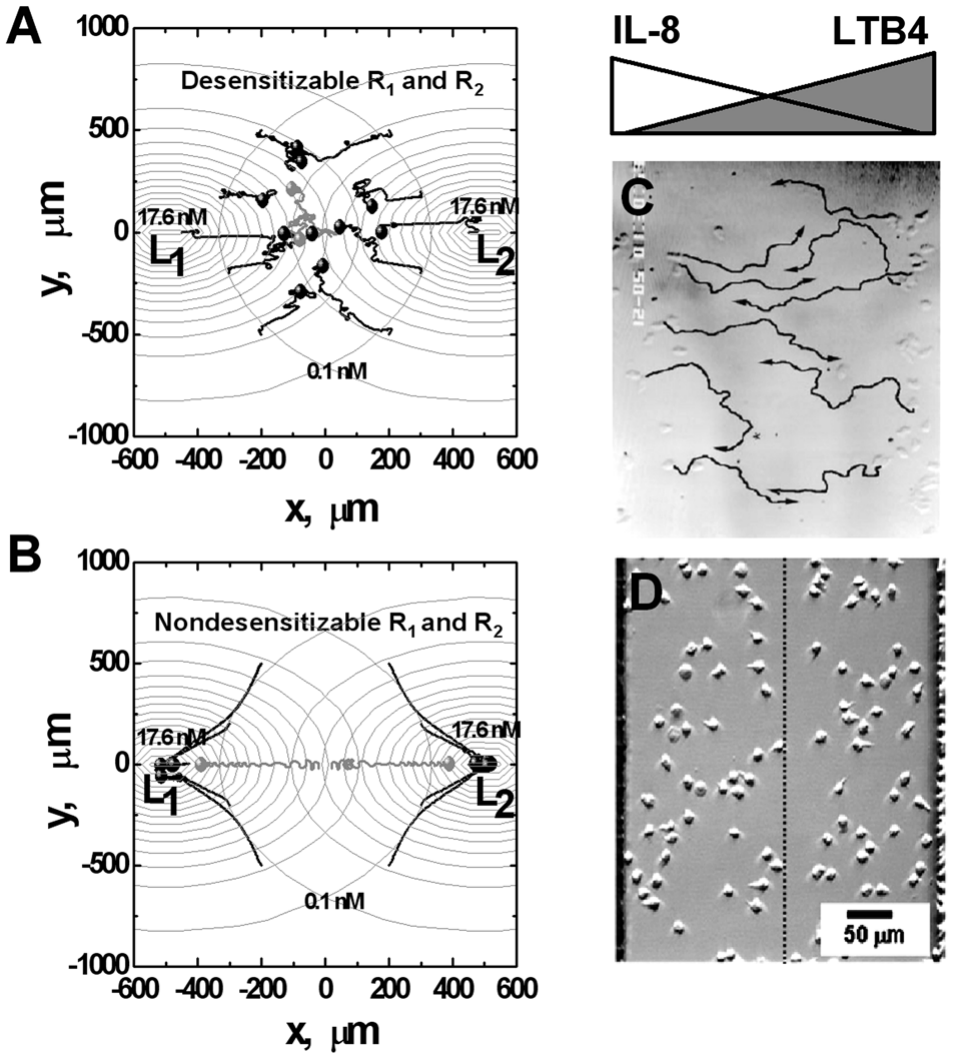
Comparison between simulation and previous experimental results of cell migration in opposing ligand gradients. (A, B) Simulation results; (C, D) Experimental results. (A) Cells expressing normal desensitizable receptors migrate toward the distant chemoattractant source. (B) In contrast, cells expressing nondesensitizable receptors migrate toward the local chemoattractant sources. The total time of simulated cell migration is 75 minutes. Twelve representative cell tracks (the starting positions of cells are consistent in (A) and (B)) are shown. The end of the tracks is indicated by solid circles. The concentration difference between adjacent circles is 1.0 nM. Grey cell tracks demonstrate the differential stability of random migration in the center zone; (C) 90 min migration tracks of neutrophils in competing gradients of IL-8 and LTB4 from experimental studies in under agarose assay [12]. Cells migrate toward the distant chemoattractant source (Reproduced from Reference 12 with the permission from The Journal of Cell Biology for noncommercial third-party use.). (D) Neutrophil migration in opposing linear gradients of IL-8 (0–6 nM/350 µm) and LTB4 (0–5.3 nM/350 µm) in a microfluidic device [16] (Reproduced from Reference 16 with the permission from Springer.). Most cells of the “left” population polarized to the right and most cells of the “right” population polarized to the left.

Computer simulations confirmed that cells preferentially sense and migrate toward the distant ligand gradient which requires receptor desensitization (Figure 5A, Movie S1 and Movie S2). The simulated migratory behaviors are in agreement with previous experimental studies of neutrophil migration in competing gradients of IL-8 and leukotriene B4 (LTB4) showing preferred migration toward the distant chemoattractant gradient (Figure 5C, 5D and Movie S3) [12], [16]. Cells expressing nondesensitizable receptors, however, migrate toward the nearer ligand source (Figure 5B and Movie S2). Comparing the cells starting from the central competing gradient region (grey tracks and circles in Figure 5A and 5B; blue tracks in Movie S1 and Movie S2), the simulations show that cells expressing normal desensitizable receptors could not migrate away from this balanced gradient region (Figure 5A and Movie S1) whereas cells expressing nondesensitizable receptors first migrate randomly and eventually leave for the nearer ligand source (Figure 5B and Movie S2). These results demonstrate the predicted receptor desensitization dependent stability of cell orientation and migration in the central region of competing gradient fields. Consistent with the orientation results, cells expressing desensitizable receptor for one ligand, but nondesensitizable receptor for the other ligand, migrate toward the nondesensitizing ligand source (data not shown).

### Cell Migration in Angled Competing Ligand Gradients

**Figure 6:**
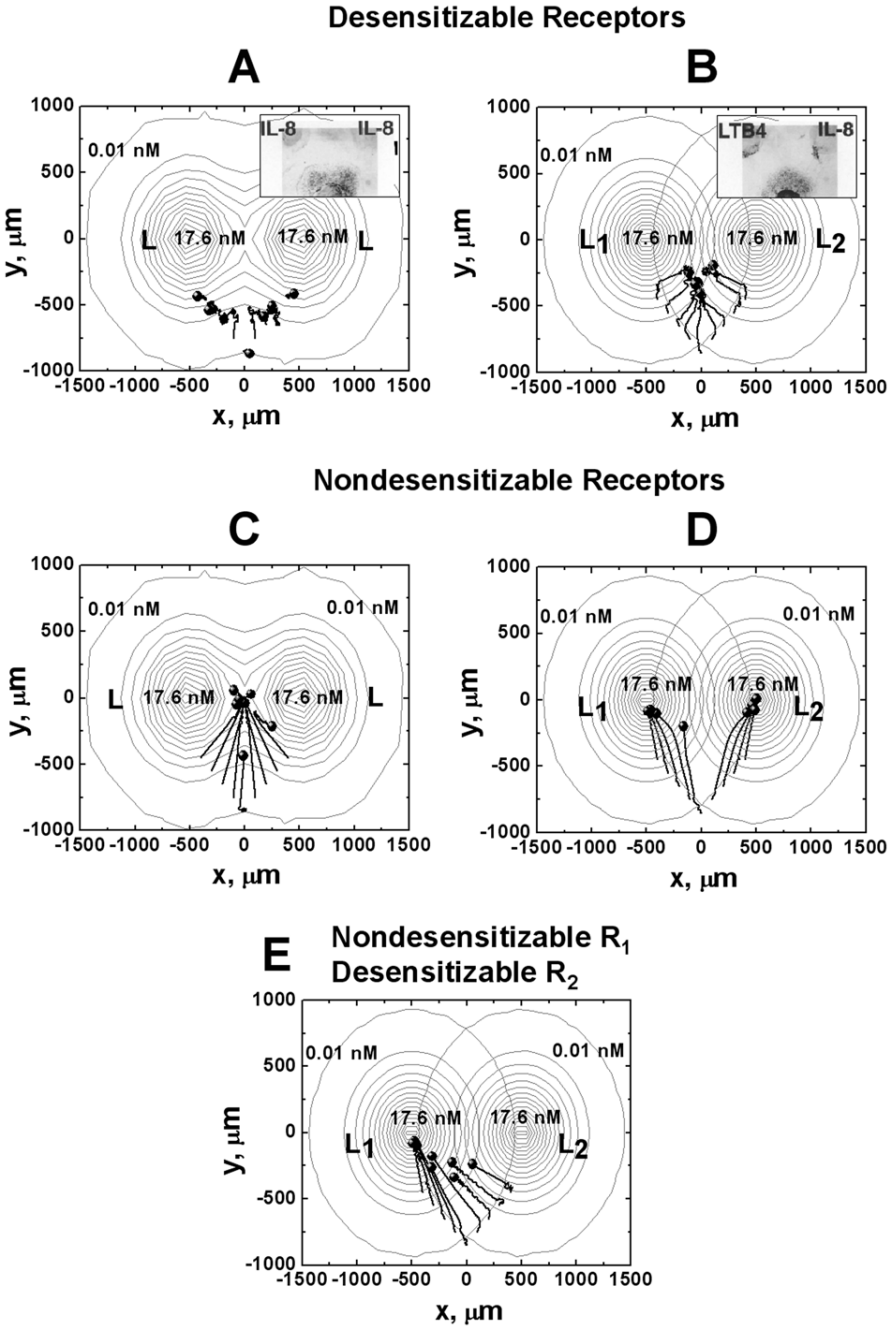
Cell migration in angled competing gradients. (A) Cells expressing desensitizable receptors migrate toward the nearer ligand source in angled competing gradients of two identical ligands; (B) In angled competing gradients of ligand *L_1_* and *L_2_*, cells expressing desensitizable receptors migrate toward the mid-point between the ligand sources of *L_1_* and *L_2_*. The inserted figures in (A, B) are previous neutrophil migration studies in under agarose assays [13] (Reproduced from Reference 13 with the permission from The Journal of Cell Biology for noncommercial third-party use.). (C) In contrast to (A), cells expressing nondesensitizable receptors migrate toward the mid-point between the two ligand sources of two identical ligands. (D) In contrast to (B), cells expressing nondesensitizable receptors migrate toward the nearer ligand sources. (E) Cells expressing nondesensitizable receptors to *L_1_* but desensitizable receptors to *L_2_*, migrate toward nondesensitizing ligand source *L_1_*. The total time of simulated cell migration is 75 minutes. Nine representative cell tracks are shown, and the starting positions of the tracks are consistent in all simulations. The end of the tracks is indicated by solid circles.

Previous neutrophil migration studies in under agarose assays show that cells can integrate multiple chemotactic signals in a vector-addition manner [13] (inserted small windows in Figure 6A and 6B). Here, the “angled gradients” are referring to the configuration that approximately the initial locations of cells and the two ligand sources form a triangle with similar side length (Figure 6). In the setting of two identical ligand sources (i.e., same ligands with same maximum concentration) with approximately equal angle to the cells, the cells migrate toward either ligand source. In contrast, cells migrate toward the midpoint of the two different ligand sources in the same geometrical gradient configuration. Our simulations of cells expressing desensitizable receptors reproduced these experimentally observed cell migration patterns with respect to angled competing gradients of two identical or different attractants (Figure 6A and 6B; and Movie S4 and Movie S5). In comparison, cells expressing nondesensitizable receptors migrate toward the midpoint of the angled competing gradient of two identical ligands, wherein a peak ligand concentration is formed by the superposition of the ligand fields from the left and the right side (Figure 6C and Movie S6). In angled competing gradients of two different ligands, nondesensitizable receptor expressing cells migrate toward the nearer ligand source (Figure 6D and Movie S7). If the cells express desensitizable receptor for one ligand, but nondesensitizable receptor for the other ligand, they migrate toward the nondesensitizing ligand source (Figure 6E and Movie S8). Thus, receptor desensitization is required for chemotactic signal integration to predict the experimentally observed cell migration pattern in angled competing chemoattractant gradients.

## Discussion

The present study further developed the previous 1-D model for cell gradient sensing to test both cell orientation and migration in 2-D single and competing ligand gradient fields with the focus on the role of ligand-induced homologous receptor desensitization. This approach adapts the previously formulated mathematical framework and parameters, but overcomes the limitations of the 1-D model to allow visualization of cell orientation and migration in 2-D gradient fields and allows comparison with experimental cell migration data. While the model is simplistic, the results are consistent with the experimentally observed preferential migration of cells toward the distant attractant source and confirm the requirement of receptor desensitization for such migratory behaviors as previously predicted by the 1-D model. Moreover, the 2-D modeling results are in qualitative agreement with the experimentally observed cell migration in angled competing ligand gradients and further show that these migration patterns are enabled by receptor desensitization. Because cell gradient sensing in the steady state and the dynamic cell migration process are fundamentally different, our study not only improves the previous modeling study to test the role of receptor desensitization in mediating cell gradient sensing in 2D, but more importantly explores the importance of this mechanism for effective migration in complex gradient fields.

Modeling of cell orientation in single 2-D ligand gradients shows that cells expressing desensitizable receptors could not sense the ligand gradient and chemotaxis toward the ligand source in the high ligand concentration zone (Figure 2A). Consistently, simulation results show decreased migration distance toward the ligand source when the maximum ligand concentration is high (Figure 3A–3C). Orientation and chemotaxis of nondesensitizable receptor expressing cells are not affected by the maximum ligand concentration of the gradient fields. Therefore, the loss of chemotactic orientation in the high ligand concentration zone and the ligand concentration dose-dependent chemotactic migration is a result of receptor desensitization by the ligand fields. These results are consistent with the well-known high dose inhibition of neutrophil orientation and chemotaxis [12], [31]. Particularly, the simulation results are in agreement with previous neutrophil migration studies showing that cells arrest at the high IL-8 concentration zone [12]. On the other hand, strong chemotaxis of nondesensitizable receptor expressing cells toward the ligand gradient predicted by our model is consistent with previous experimental studies showing comparable or even higher levels of chemotaxis of cells mediated by nondesensitizable receptor mutants in either transfected cell models [18], [19] or leukocytes from patients with Warts, hypogammaglobulinemia, infections, and myelokathexis (WHIM) syndrome [32]. Interestingly, experimental studies have shown that chemokine CCL21 alone is sufficient to attract T cells and dendritic cells to the secondary lymphoid tissues in the absence of CCL19 [33]. In this context, the lack of ability of CCL21 for desensitizing chemokine receptor CCR7 expressed on T cells and dendritic cells may be taken into account and can be explained by our model. There is currently no experimental data available for the role of receptor desensitization in cell gradient sensing and migration in competing gradient fields. However, the experimental systems as described above (i.e. cells expressing transfected mutant receptors; chemokine CCL21 that desensitizes its receptor CCR7 at low level; leukocytes from WHIM syndrome patients.) can be potentially used to test the modeling predictions in competing gradient fields.

2-D modeling and simulations clearly show that the preferred orientation and migration of cells toward the distant ligand gradient over the local competing ligand gradient requires receptor desensitization. Receptor desensitization allows cells to effectively reach an intermediate zone between the two competing ligand sources wherein the competing signals are balanced. Because of the stability of this random migration zone, cells exhibit chemokinesis without being attracted away by the ligand sources. In contrast, cells expressing nondesensitizable receptors have difficulties to enter and stay in this zone due to the strong nondesensitizing chemotactic signals from the ligand sources and the instability of the center zone for randomly migration. As discussed in the previous 1-D model, such balanced chemotactic migration provides a mechanism for maximizing interactions between antigen presenting cells (APCs) and T cells by attracting the two cell populations to the junction between the chemokine-defined domains within secondary lymphoid tissues [8].

**Figure 7:**
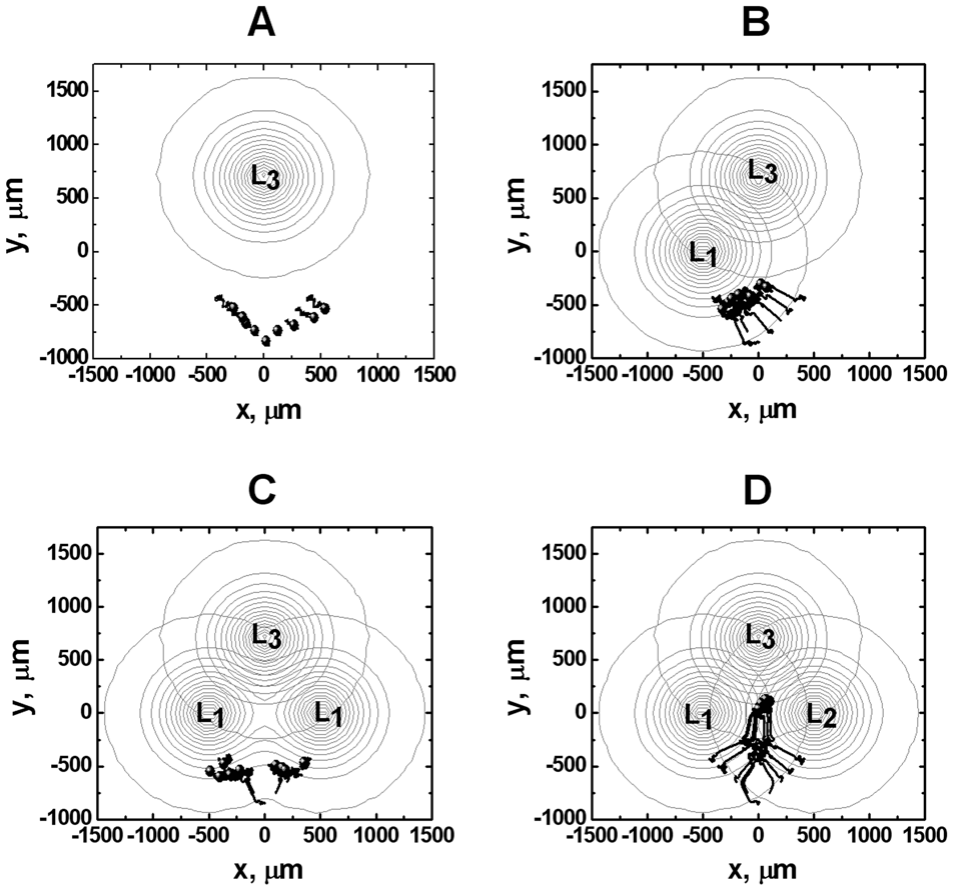
Comparison of strategies for directing cells to the distant target as shown by simulation of cell migration in different ligand gradient configurations. (A) Single ligand gradient from the distance could not reach and attract cells. (B) The distant target *L_3_* combined with the closer ligand *L_1_* could not effectively attract cells further toward the distant target as the cell may reach the high-dose saturation region of *L_1_* field before it senses *L_3_* gradient. (C) The distant target *L_3_* combined with angled competing gradients of two identical ligands *L_1_* could not effectively attract cells further toward the distant target. (D) The distant target *L_3_* combined with angled competing gradients of two different ligands *L_1_* and *L_2_* effectively attract cells toward the distant target. The total time of simulated cell migration is 150 minutes. Nine representative cell tracks are shown, and the starting positions of the tracks are consistent in all simulations. The end of the tracks is indicated by solid circles.

The 2-D model allows examination of cell migration in different configurations of competing ligand gradients in a 2-D plane. In angled competing gradients of two identical ligands, a superimposed single ligand field is produced that attracts cells to either ligand source depending on the cell's initial position. Because of high-dose inhibition of chemotaxis as discussed previously, migration distance of cells toward the desensitizing ligand gradients is limited (Figure 3A–3C, Figure 6A, Movie S4). In contrast, in angled competing gradients of two different ligands, cells are able to effectively integrate the competing signals and migrate toward and reach the center zone of the gradient fields (Figure 6B and Movie S5). In this zone, the attracting signals from the two ligands are balanced and thus the net chemotactic signal is below the threshold or at a minimum. If a third ligand gradient from the distance is presented to the cells in this zone, the cells are predicted to migrate toward the new ligand gradient. As demonstrated by simulations of cell migration in Figure 7, comparing to the configurations of only one or two spatially arranged ligand gradient fields (Figure 7A–C. in these configurations, cells cannot migrate to the region, wherein the distant ligand gradient can be detected.), combinatorial guidance by multiple different attractants (Figure 7D) has the advantage of directing cells to the target from a distance. Thus, our receptor desensitization model accounts for the hypothesized multi-step chemotactic navigation model for cells to reach the distant target [12], [13], and argues the importance of the multiple different chemoattractants based guiding mechanism. On the other hand, nondesensitizable receptors mediate strong chemotaxis of cells to the ligand sources in angled competing ligand gradients (Figure 6C–6E; and Movie S6, S7, S8). This mechanism allows nondesensitizing chemokines such as CCL21 to robustly attract leukocytes to secondary lymphoid tissues through their interacting receptor such as CCR7 signaling without distraction from other desensitizing chemokines [33]. Although cells can reach the center zone of angled competing gradients of the same nondesensitizing ligand (Figure 6C and Movie S6), they will not be able to further move toward the third desensitizing ligand source due to the strong local nondesensitizing chemotactic signals. A moving chemoattractant source may attract the cell over a long distance. However, such a strategy requires the moving chemoattractant source to be initially in the cell detection range and moves at low speed relative to the cell (File S1).

Similar to the analysis in the previous 1D model [25], we performed parametric analysis to test the model in single and competing ligand gradients for a range of values for different kinetic rates (Figure 2C–2E and Figure 4D&4E). Our results demonstrate the dependence of cell orientation on the three important ligand-induced receptor modulation rates (*k_des_*, *k_i_* and *k_up_*) and on the relative cell positions in the gradient fields, suggesting that the receptor desensitization mechanism is one important mechanism but certainly not the only mechanism for effective cell gradient sensing and migration in simple and complex ligand fields.

The current simple 2-D model has the potential to be further developed to consider the downstream signaling pathways such as G-protein signaling for mediating cell gradient sensing and migration. As discussed in the Model section, different ligands and receptors can in principle have different recycling kinetics and can trigger different downstream chemotactic pathways [28], [29] and this effect can be modeled by further developing the current model. Indeed, previous studies have shown a signaling hierarchy between the end-target-derived chemoattractants and tissue-derived chemoattractants, each trigger a different downstream chemotactic signaling pathway [15], [34]. On the other hand, our current model focuses on tissue-derived chemoattractants and assumes common kinetic properties and downstream signaling pathways for the two ligand-receptor pairs, and the results are in qualitative agreement with experimental results of neutrophil migration in competing IL-8 and LTB4 gradients. In addition, the current model applies fixed ligand gradients with identical shape for both ligands to the cells. Future work will consider different time-evolving ligand gradients to better mimic the dynamic chemoattractant fields in tissues. For example, previous neutrophil chemotaxis studies show that the addition of a second low dose attractant gradient after cells arrested by the first high dose attractant gradient allows cells to continue to migrate toward the second gradient [12]. Such migratory responses can be modeled with dynamic gradient configurations. Moreover, the effect of receptor drifting on the cell surface, the differential receptor recycling in different parts of the cell and more complex cell morphology can be considered to further develop the model. Finally, further modeling of cross receptor desensitization by competing ligand gradients will provide more insights into the receptor desensitization mediated chemotactic guiding mechanisms for cell gradient sensing and cell migration. In summary, the current 2-D model accounts for the experimental cell migration data in single and competing attractant gradients which supports the hypothesized receptor desensitization mechanism behind the multi-step chemotactic navigation model. Furthermore it provides interesting insights into chemoattractant guided leukocyte trafficking to and positioning within secondary lymphoid tissues.

## Supporting Information

File S1 **Supporting simulation data and parameter justification.**
(DOCX)

Movie S1 **Computer simulation of the migration of normal cells in opposing gradients of L_1_ and L_2_.** Cells expressing normal desensitizable receptors (i.e. normal cells) migrate toward the distant ligand source. Blue cell tracks demonstrate the stability of random migration in the center zone of the gradient fields. The total time of simulated cell migration is 75 minutes.
(MOV)

Movie S2 **Computer simulation of the migration of mutant cells in opposing gradients of L_1_ and L_2_.** In contrast to Movie S1, cells expressing nondesensitizable receptors (i.e. mutant cells) migrate toward the local ligand source. Blue cell tracks demonstrate that cells starting from the center zone of the gradient fields first migrate randomly and eventually leave for the nearer ligand source. The total time of simulated cell migration is 75 minutes.
(MOV)

Movie S3 **Experimental results of cell migration in opposing gradients of IL-8 and LTB4.** Neutrophils migrate in opposing linear gradients of IL-8 (0–6 nM/350 µm) and LTB4 (0–5.3 nM/350 µm) (Reproduced with the permission from Francis Lin (Ref. 16).). Most cells of the “left” population migrated to the right and most cells of the “right” population migrated to the left.
(MOV)

Movie S4 **Computer simulation of the migration of normal cells in angled gradients of ligand L.** Cells expressing normal desensitizable receptors (i.e. normal cells) migrate toward the nearer ligand source in angled gradients of ligand L depending on the cells' initial positions relative to the ligand sources. The inserted picture is from previous neutrophil migration studies in angled gradients of IL-8 using under agarose assays (Reproduced from Reference 13 with the permission from The Journal of Cell Biology for noncommercial third-party use.). The total time of simulated cell migration is 75 minutes.
(MOV)

Movie S5 **Computer simulation of the migration of normal cells in angled gradients of L_1_ and L_2_.** In angled competing gradients of two different ligands, cells expressing normal desensitizable receptors (i.e. normal cells) migrate toward the mid-point between the two ligand sources. The inserted picture is from previous neutrophil migration studies in angled competing gradients of IL-8 and LTB4 using under agarose assays (reproduced from Reference 13 with the permission from The Journal of Cell Biology for noncommercial third-party use). The total time of simulated cell migration is 75 minutes.
(MOV)

Movie S6 **Computer simulation of the migration of mutant cells in angled gradients of ligand L.** In contrast to Movie S4, cells expressing nondesensitizable receptors (i.e., mutant cells) migrate toward the mid-point between the two ligand sources. The total time of simulated cell migration is 75 minutes.
(MOV)

Movie S7 **Computer simulation of the migration of mutant cells in angled gradients of L_1_ and L_2_.** In contrast to Movie S5, cells expressing nondesensitizable receptors (i.e. mutant cells) migrate toward the nearer ligand sources depending on the cells' initial positions relative to the ligand sources. The total time of simulated cell migration is 75 minutes.
(MOV)

Movie S8 **Computer simulation of the migration of mutant cells specific to L_1_ in angled gradients.** In angled gradients of L_1_ and L_2_, cells expressing nondesensitizable receptors to L_1_ but normal desensitizable receptors to L_2_ (i.e. mutant cells specific to L_1_), migrate toward nondesensitizing ligand source L_1_. The total time of simulated cell migration is 75 minutes.
(MOV)
