# Supplementary material for: Modeling Cell Gradient Sensing and Migration in Competing Chemoattractant Fields
Source: PLoS One. 2011 Apr 29;6(4):e18805. doi: 10.1371/journal.pone.0018805 (PMC3084714; doi:10.1371/journal.pone.0018805)
Supplement: File S1 — Supporting simulation data and parameter justification. (DOCX) [file pone.0018805.s001.docx]

**Modeling Cell Gradient Sensing and Migration in Competing Chemoattractant Fields**

Dan Wu and Francis Lin

**Supporting Information S1**

**Figure S1.**

**Figure S1.** **Fitting of the experimentally measured gradient and the calculated diffusion gradient profile by power function.** (A) The gradient profile of LTB4 in the under agarose assay at 30min (Foxman, et al, J Cell Biol, 1997) can be effectively fitted by the power function (*y=a+bx^n^*) with the power n=3.35. The value of reduced χ^2^ is 0.001. (B) The calculated gradient profile from fixed point-source free diffusion can be effectively fitted by the power function (*y=a+bx^n^*) with the power n=2.9 at t=3min for 10kD chemokine molecules in medium. The value of reduced χ^2^ is 0.92. Note that the highest concentration for both the calculated diffusion gradient profile and the experimentally measured LTB4 gradient are considered in the right side of gradient field.

**Figure S2.**

**Figure S2. Simulated migration of cells expressing normal desensitizable receptors in slow moving single ligand gradients.** The velocity of moving gradient is v = 0.1 μm/s to the rightward direction. The total time of simulated cell migration is 450 minutes and the tracks of cells are shown at (A) 0 minute; (B) 150 minute; (C) 300 minute; (D) 450 minute. Seven representative cell tracks are shown and the end of the tracks is indicated by solid circles. The ligand gradient is represented by contour plot with the highest ligand concentration (20 nM) at the center of the contours. The simulation results show that cells can follow the slow moving ligand gradient over a long distance.

**Figure S3.**

**Figure S3. Simulated migration of cells expressing normal desensitizable receptors in fast moving single ligand gradients.** The velocity of moving gradient is v = 0.2 μm/s to the rightward direction. The total time of simulated cell migration is 450 minutes and the tracks of cells are shown at (A) 0 minute; (B) 150 minute; (C) 300 minute; (D) 450 minute. Seven representative cell tracks are shown and the end of the tracks is indicated by solid circles. The ligand gradient is represented by contour plot with the highest ligand concentration (20 nM) at the center of the contours. The simulation results show that cells cannot effectively follow the fast moving ligand gradient.
